# Radiomics of dynamic contrast-enhanced magnetic resonance imaging parametric maps and apparent diffusion coefficient maps to predict Ki-67 status in breast cancer

**DOI:** 10.3389/fonc.2022.847880

**Published:** 2022-11-25

**Authors:** Shuqian Feng, Jiandong Yin

**Affiliations:** ^1^ Department of Radiology, Shengjing Hospital of China Medical University, Shenyang, Liaoning, China; ^2^ School of Intelligent Medicine, China Medical University, Shenyang, Liaoning, China

**Keywords:** breast cancer, radiomics, dynamic contrast-enhanced magnetic resonance imaging, apparent diffusion coefficient, Ki-67

## Abstract

**Purpose:**

This study was aimed at evaluating whether a radiomics model based on the entire tumor region from breast dynamic contrast-enhanced magnetic resonance imaging (DCE-MRI) parametric maps and apparent diffusion coefficient (ADC) maps could indicate the Ki-67 status of patients with breast cancer.

**Materials and methods:**

This retrospective study enrolled 205 women with breast cancer who underwent clinicopathological examination. Among them, 93 (45%) had a low Ki-67 amplification index (Ki-67 positivity< 14%), and 112 (55%) had a high Ki-67 amplification index (Ki-67 positivity ≥ 14%). Radiomics features were extracted from three DCE-MRI parametric maps and ADC maps calculated from two different b values of diffusion-weighted imaging sequences. The patients were randomly divided into a training set (70% of patients) and a validation set (30% of patients). After feature selection, we trained six support vector machine classifiers by combining different parameter maps and used 10-fold cross-validation to predict the expression level of Ki-67. The performance of six classifiers was evaluated with receiver operating characteristic (ROC) analysis, sensitivity, and specificity in both cohorts.

**Results:**

Among the six classifiers constructed, a radiomics feature set combining three DCE-MRI parametric maps and ADC maps yielded an area under the ROC curve (AUC) of 0.839 (95% confidence interval [CI], 0.768−0.895) within the training set and 0.795 (95% CI, 0.674−0.887) within the independent validation set. Additionally, the AUC value, compared with that for a single parameter map, was moderately increased by combining features from the three parametric maps.

**Conclusions:**

Radiomics features derived from the DCE-MRI parametric maps and ADC maps have the potential to serve as imaging biomarkers to determine Ki-67 status in patients with breast cancer.

## Introduction

Breast cancer (BC) is the most prevalent malignant tumor type threatening women’s health globally (1). According to an immunohistochemistry (IHC) classification system, BC can be divided into four subtypes, basal-like, HER2-enriched, and luminal A and B subtypes, on the basis of the expression of progesterone receptor (PR), estrogen receptor (ER), human epidermal growth factor receptor 2 (HER2), and Ki-67 (2). Ki-67 protein is a recognized marker of tumor proliferation and invasiveness (3), as well as a recognized indicator of BC prognosis (4). Ki-67 can be used as a molecular marker to distinguish the molecular subtypes of luminal A and B (5). A high expression level of Ki-67 is associated with poorer prognosis (3, 6), greater risk of recurrence (7), and worse survival outcomes (8). Hence, accurately identifying the status of the Ki-67 index is crucial for the prognosis of BC.

Dynamic contrast-enhanced magnetic resonance imaging (DCE-MRI) is useful for assessing tumor anatomical information and angiogenesis (9). Radiomics involves high-throughput extraction of many image-based features from standard medical images and determining the potential links between these features and pathophysiology (10, 11). Radiomics analysis of features extracted from DCE-MRI images can be used to distinguish the HER2 2+ status, predict lymphovascular invasion, determine the status of lymph node metastasis, and identify the degree of tumor malignancy (12–19). The apparent diffusion coefficient (ADC), a quantitative parameter generated in diffusion-weighted imaging (DWI), is the most used clinical parameter reflecting the degree of tissue distribution according to the diffusion of water molecules (20). Since ADC is influenced by cell density and tissue structure, Choi (12) proposed that DCE-MRI combined with DWI is helpful to evaluate the status of lymphovascular invasion in patients with node-negative invasive BC. In addition, ADC values have been shown to correlate with the Ki-67 index (20, 21). Therefore, radiomics analysis based on DCE parameters and ADC might have the potential to predict Ki-67 status and even improve predictive performance.

A previous study has shown that the radiomics features derived from DCE-MRI functional parameter maps achieved the best results in identifying sentinel lymph node metastasis status in patients with BC (22). Another study has predicted the Ki-67 index and HER2 2+ status by using intratumoral and peritumoral radiomics features based on six DCE-MRI functional parameter maps (14). Both of these studies used single-layer lesions and consequently might have overlooked the correlations between layers. Jong et al. have investigated the correlation between quantitative MR parameters and Ki-67 expression status by analyzing DCE-MRI and DWI sequences in ER-positive invasive BC (23). However, their analysis of the interstitial signal enhancement ratio used only univariate and multivariate analysis, without radiomics analysis. To our knowledge, few studies have used a combination of breast MRI functional parametric maps and ADC maps in radiomics analysis. Moreover, in most prior studies, region of interest (ROI) depiction has been performed primarily on the slice images showing the largest tumor size (24–26). In this study, radiomics features were extracted from the entire tumor volume on the basis of three semi-quantitative parametric maps and ADC maps, and the predictive performance of the classification models based on three-dimensional features in terms of Ki-67 expression status was evaluated.

Therefore, the purpose of our study was to evaluate the performance of a radiomics model based on the entire tumor region from three DCE-MRI parametric maps and ADC maps to determine the status of Ki-67 in patients with BC.

## Materials and methods

### Study population

This research was approved by the ethics committee of our institution. Given the retrospective nature of the study, the requirements for informed consent were waived.

**Figure 1:**
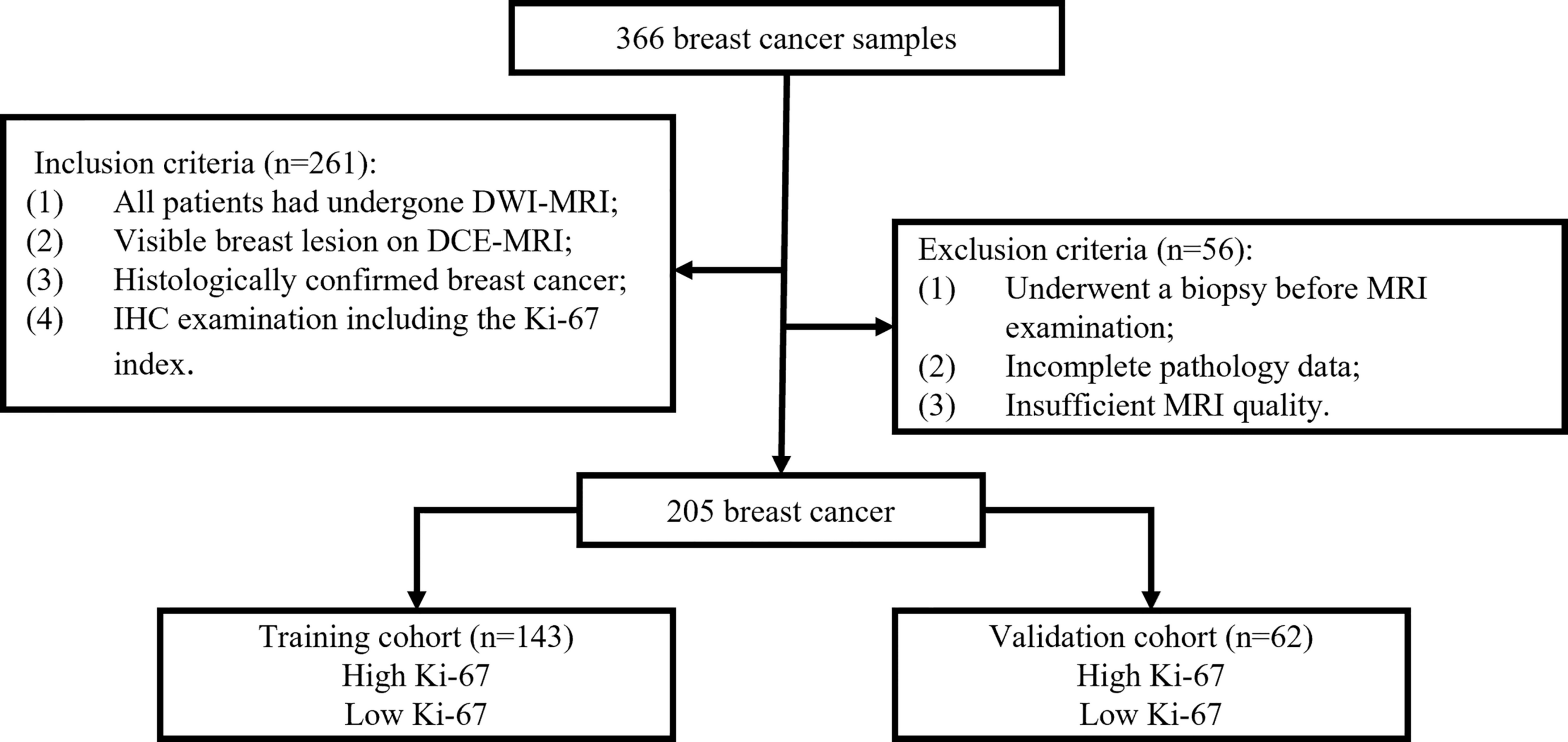
Flowchart of the patient recruitment process in this study.

Between December 2018 and September 2020, 366 patients with pathologically confirmed primary BC who underwent breast DCE-MRI at Shengjing Hospital were enrolled in the study. **Figure 1** shows the patient recruitment process for this study. The inclusion criteria were as follows. All included patients 1) underwent DWI-MRI, 2) had clear breast lesions on magnetic resonance images, 3) had BC confirmed through histologic examination, and 4) underwent IHC examination, including the Ki-67 index. The exclusion criteria were as follows. The excluded patients 1) underwent biopsy before MRI, 2) had incomplete pathological data, or 3) had insufficient MRI quality because of clear motion artifacts.

**Figure 2:**
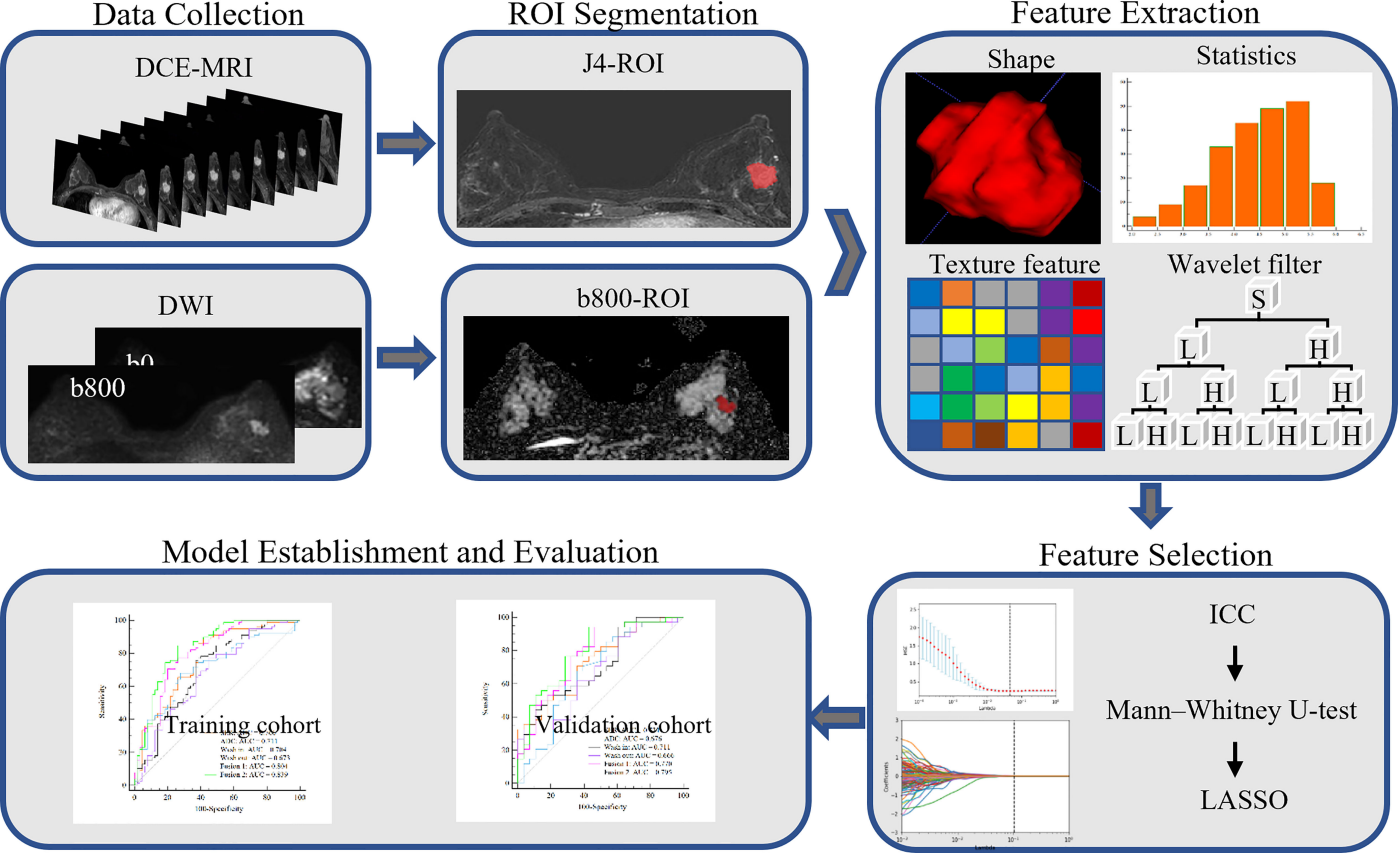
Framework for the radiomics workflow.

The final cohort consisted of 205 patients who were randomly divided into a training set and a validation set, in proportions of 70% and 30%, respectively. The training dataset (n = 143) comprised 65 patients with low Ki-67 expression and 78 patients with high Ki-67 expression. The validation dataset (n = 62) comprised 28 and 34 patients with low and high Ki-67 expression, respectively. The clinical characteristics of all patients are described in **Table 1**, and the framework for the radiomics workflow is shown in **Figure 2**.

**Table 1:** Clinicopathological characteristics according to Ki-67 proliferation status.

Variables	Total (n = 205)	Low-Ki-67 (n = 93)	High-Ki-67 (n = 112)	*p*-Value^a^
**Age, mean ± SD, years**	50.56 ± 9.7	49.9 ± 9.9	51.1 ± 9.6	0.394
**Tumor size, mean ± SD, mm**	27.60 ± 15.2	25.4 ± 15.5	29.4 ± 14.9	0.061
**ER status** ^c^				<0.05^b^
Negative	35 (17.1%)	9 (9.7%)	26 (23.2%)	
Positive	170 (82.9%)	84 (90.3%)	86 (76.8%)	
**PR status** ^c^				<0.05^b^
Negative	51 (24.9%)	11 (11.8%)	40 (64.3%)	
Positive	154 (75.1%)	82 (88.2%)	72 (35.7%)	
**HER2 status** ^c^				<0.05^b^
Negative	141 (68.8%)	80 (86.0%)	61 (54.5%)	
Positive	64 (31.2%)	13 (14.0%)	51 (45.5%)	
**Histological type** ^d^				<0.05^b^
Invasive ductal carcinoma	191 (93.2%)	79 (84.9%)	112 (100%)	
Other	14 (6.8%)	14 (15.1%)	0 (0.0%)	
**Histological grade** ^d^				<0.05^b^
I	13 (6.3%)	13 (14.0%)	0 (0.0%)	
II	162 (79.0%)	70 (75.3%)	92 (82.1%)	
III	30 (14.7%)	10 (10.7%)	20 (17.9%)	
**TIC type** ^d^				0.068
Plateau	51 (24.9%)	30 (32.3%)	21 (18.8%)	
Wash-out	150 (73.2%)	62 (66.7%)	88 (78.6%)	
Wash-in	4 (1.9%)	1 (1.0%)	3 (2.6%)	

SD, standard deviation; ER, estrogen receptor; PR, progesterone receptor; HER2, human epidermal growth factor receptor 2; TIC, time-intensity curve.

a p-Value comparing low Ki-67 to high Ki-67.

b p< 0.05 is considered statistically significant.

c Data were tested with the chi-square test.

d Data were tested with Fisher’s exact test.

### Pathological assessment

Streptavidin peroxidase IHC was used to detect the expression levels of ER, PR, HER2, and Ki-67 in each patient. If at least 1% of the tumor nuclei were ER or PR positive, the ER or PR status was determined to be positive (27). A Ki-67 proliferation index ≥14% was considered high, and a value<14% was considered low (28). HER2 status was considered positive when the HER2 staining intensity score was 3+ and negative when the score was 0 or 1+. If the HER2 staining intensity score was 2+, and further fluorescence *in situ* hybridization confirmed gene amplification, the result was considered positive (29).

### MR image acquisition

DCE-MRI was performed at 3.0 T with a Signa HDxt 3.0 T MRI scanner (GE Healthcare Life Sciences, Chicago, IL, USA). All patients were scanned in a prone position with a dedicated eight-channel double-breast coil. Axial DWI sequence scanning was performed before DCE-MRI acquisition. The acquisition parameters were as follows: repetition time, 4,000 ms; flip angle, 90°; echo time, 83.30 ms; field of view, 340 × 340 mm2; matrix size, 256 × 256; slice thickness, 4.50 mm; spacing between slices, 5.00 mm; and b values, 0 and 800 s/mm^2^. The ADC maps were calculated from diffusion images with two b values.

Second, an axial fat-saturated T1-weighted pre-contrast scan based on the VIBRANT-VX technique was acquired. After the intravenous injection of a contrast agent (Magnevist, Bayer Healthcare Pharmaceuticals, Berlin, Germany) at 4 ml/s with a dose of 0.15 mmol per kg body weight, eight post-contrast scans were acquired with the following parameters: repetition time, 4.14 ms; flip angle, 12°; echo time, 2.10 ms; slice thickness, 2.00 mm; spacing between slices, 1.00 mm; and field of view, 340 × 340 mm^2^. Finally, eight subtraction sequences were obtained through the subtraction of each pre-contrast scan sequence from the eight post-contrast scan sequences.

### Tumor segmentation

Tumor segmentation must be completed before the extraction of high-throughput quantitative features. We used ITK-SNAP software to perform three-dimensional manual segmentation (open-source software; www.itk-snap.org). Two radiologists with 8 years (reader 1) and 10 years (reader 2) of experience in breast MR imaging diagnosis completed the layer-by-layer manual segmentation of tumor area in the MR images.

DCE-MRI images in all cases were segmented on the fourth subtraction sequence, which is usually useful for visual examination because it is usually the most enhanced among all time series (30). For the ADC map, the tumor contour was manually drawn along the boundary of the high signal area on each transverse DWI slice (b value of 800 s/mm^2^) (21). After manual sketching, the software automatically generated the three-dimensional tumor volume model, which was finally copied to the corresponding ADC maps.

### Parametric map generation

Before the extraction of radiomics features, three functional parameter maps and ADC maps were calculated pixel by pixel according to the following formula.

Wash-in maps:


(1) $$((SI_{1}\;–\;SI_{0})\;/SI_{0})\;\times \;\mathrm{100}\%$$

Wash-out maps:


(2) $$((SI_{1}\;–\;SI_{8})\;/SI_{1})\;\times \;\mathrm{100}\%$$

Signal enhancement ratio (SER) maps:


(3) $$((SI_{1}\;–\;SI_{0})/(SI_{8}\;–\;SI_{0}))\;\times \;\mathrm{100}\%$$

**Figure 3:**
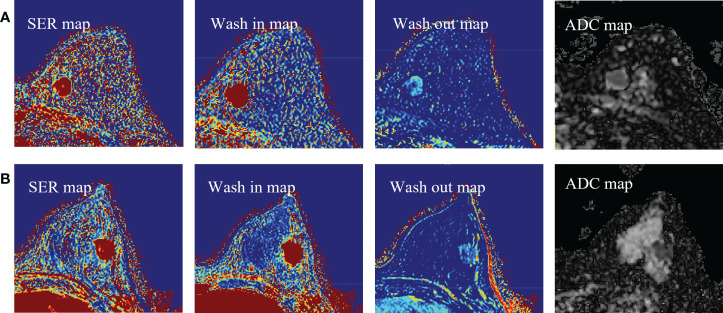
**(A)** Representative images of DCE-MRI parametric maps and ADC maps of low Ki-67 status. **(B)** Representative images of DCE-MRI parametric maps and ADC maps of high Ki-67 status. DCE-MRI, dynamic contrast-enhanced magnetic resonance imaging; ADC, apparent diffusion coefficient.

ADC maps:


(4) $$\left(InSI_{b0}\;–\;InSI_{b800})/(b–b0\right)$$

where SI is the signal intensity of each pixel in the image, SI_0_ represents the value of the pixel in the pre-contrast image, SI_1_ and SI_8_ represent the pixel value in the first and eighth post-contrast scans, and SI_b0_ and SI_b800_ represent the signal intensity when the b value of the DWI sequence is 0 and 800 s/mm^2^, respectively. Representative images of DCE-MRI parametric maps and ADC maps are shown in **Figure 3**.

### Radiomics feature extraction

Feature extraction was performed with an in-house texture extraction platform developed with the Python (3.6.2) package PyRadiomics (31). A total of 946 radiomics features were extracted from each map. These features included 86 original features (consisting of five categories of features: first-order statistics, gray-level co-occurrence matrix, gray-level run-length matrix, gray-level size zone matrix, and gray-level dependence matrix), 172 Laplacian of Gaussian (sigma = 3.0, 5.0 mm) features, and 688 wavelet features (also composed of five categories of features). After the addition of eight shape features from DCE-MRI, we ultimately obtained 3,792 radiomics features from the SER maps, wash-out maps, wash-in maps, and ADC maps. The details of the extracted features are shown in **Supplementary Table 1**.

### Feature selection and radiomics model construction

Prior to feature selection, we calculated the intra-class correlation coefficients (ICCs) to evaluate the reproducibility and stability of radiomics features extracted from segmented images performed by two experienced radiologists. Features with a good consistency (ICC > 0.8) were retained for further radiomics analysis.

For feature selection, significant radiomics features with *p*< 0.05 between patients with high versus low Ki-67 expression were first identified with the Mann–Whitney U-tests through a backward selection approach. Second, the remaining features were normalized separately with the Z-score to make the dynamic ranges comparable. Subsequently, the least absolute shrinkage and selection operator (LASSO) logistic regression method, which is suitable for dimensionality reduction of high-dimensional data, was used to select the radiomics features of the training data. To avoid overfitting, the optimal value of the LASSO regularization parameter lambda was determined through 10-fold cross-validation. Finally, the most important features obtained in LASSO selection were used to establish support vector machine classifiers to predict the Ki-67 proliferation status in BC, wherein the kernel parameter was set as a linear kernel, and the other parameters were set as default values (32).

Feature selection and machine learning classifier construction were executed in Python software (version 3.6.2, Welcome to Python.org).

### Statistical analysis

The statistical differences in age and tumor size between groups with high and low Ki-67 expression were evaluated with independent-samples *t*-tests. Differences in categorical variables between the molecular subtype characteristics were evaluated with chi-square tests. If the expected frequency of any cell in the table was less than five, it was tested with Fisher’s exact test. Receiver operating characteristic (ROC) curves were drawn with the optimal threshold determined by the maximum Youden index. The area under the ROC curve (AUC) and the classification sensitivity and specificity in the training and validation groups were calculated to predict the Ki-67 status. The AUC between the two models in the validation set was statistically compared using DeLong’s test. Statistical analysis was performed in SPSS software (version 23.0, Chicago, IL, USA). Professional statistical software MedCalc (version 20.0.3, https://www.medcalc.org/) was used to construct the ROC curves.

## Results

### Patient characteristics

The statistical test results of the correlations between molecular subtypes and pathological and clinical characteristics are listed in **Table 1**. We observed no significant difference in mean age, mean maximum tumor diameter, or dynamic enhancement time-intensity curve type between the groups with high or low Ki-67 expression (*p* = 0.394, 0.061, and 0.068). However, we did observe significant differences in ER status, PR status, HER2 status, pathologic type, and pathologic grade (*p*< 0.05).

### Radiomics model construction and assessment of performance

Among the 3,792 radiomics features initially extracted, 2,622 (69.1%) had good interobserver consistency (ICCs > 0.8) and were included in further analysis.

**Figure 4:**
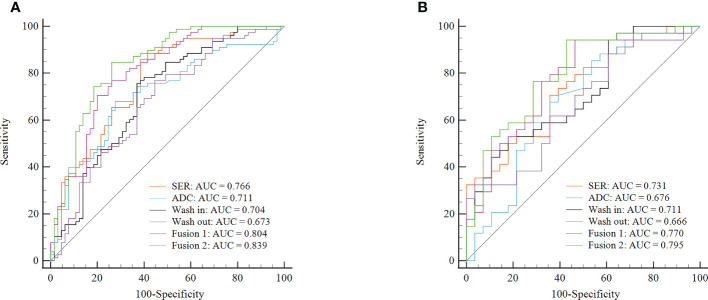
ROC curves of six classifiers for identification of Ki-67 status in each cohort. **(A)** ROC curves of classifiers for identification of Ki-67 status in the training cohort. **(B)** ROC curves of classifiers for identification of Ki-67 status in the validation cohort. Fusion 1 represents the SVM model established by combining radiomics features from three DCE-MRI parameter maps (SER, wash-in, and wash-out). Fusion 2 represents the SVM model established by combining radiomics features from three DCE-MRI parameter maps and ADC maps. ROC, receiver operating characteristic; SVM, support vector machine; SER, signal enhancement ratio.

To perform Ki-67 status recognition, five, four, six, and one features were selected from the wash-out, wash-in, SER, and ADC maps, respectively, and 14 and 15 features were selected from two combined parameter maps (DCE-MRI parameter maps and DCE-MRI combined with ADC maps, respectively). The details of the 15 features selected from the combined DCE-MRI and ADC maps are shown in **Table 2**. The feature details selected from other maps are shown in **Supplementary Tables 2**–**5**. We then established six support vector machine classifiers to predict Ki-67 status according to the final retained features. The performance of the classifiers was evaluated on the basis of ROC curves, and the results are presented in **Figure 4**. Classifiers containing features extracted from a wash-out, wash-in, SER, and ADC maps were evaluated. After the addition of the features of ADC maps, the performance of the model combined with the three parameter maps improved, and the AUC value was 0.839 (95% CI, 0.768, 0.895) in the training set and 0.795 (95% CI, 0.674, 0.887) in the validation set (**Table 3**). In addition, compared with that of the single parameter image, the predictive performance of the support vector machine (SVM) model in the training dataset was significantly improved by combining the features of each map (i.e., wash-out, wash-in, SER, and ADC maps) (*p*< 0.001; *p* = 0.001, 0.001, 0.002). The performance of the combined model in the validation data set was higher than that of the model based on the SER parameter maps (*p* = 0.040; **Table 4**).

**Table 2:** Description of the selected radiomics features from DCE-MRI combined with ADC maps.

Different map	Radiomics feature	Radiomics group	Feature class filter
**SER**	Dependence non-uniformity normalized	Gldm	Log-sigma-3.0 mm
**SER**	Dependence variance	Gldm	Log-sigma-3.0 mm
**SER**	Minimum	First order	Log-sigma-5.0 mm
**SER**	Tenth percentile	First order	Wavelet-LLH
**SER**	Run variance	Glrlm	Wavelet-LHL
**SER**	Autocorrelation	Glcm	Wavelet-LHH
**Wash-in**	Minimum	First order	Log-sigma-3.0 mm
**Wash-in**	Large area high gray-level emphasis	Glszm	Log-sigma-5.0 mm
**Wash-in**	range	First order	Wavelet-LHH
**Wash-in**	Small dependence high gray-level emphasis	Gldm	Wavelet-LHH
**Wash-in**	Large dependence low gray-level emphasis	Gldm	Wavelet-HHL
**Wash-out**	Size zone non-uniformity normalized	Glszm	Log-sigma-3.0 mm
**Wash-out**	Correlation	Glcm	Log-sigma-3.0 mm
**Wash-out**	Joint energy	Glcm	Wavelet-LLL
**ADC**	Gray-level non-uniformity	Gldm	Original

SER, signal enhancement ratio; ADC, apparent diffusion coefficient; Gldm, gray-level dependence matrix; Glrlm, gray-level run length matrix; Glcm, gray-level co-occurrence matrix; Glszm, gray-level size zone matrix; LoG, Laplacian of Gaussian.

**Table 3:** Predictive performance of six models in the training and validation cohorts.

Model	Training			Validation		
	AUC (95% CI)	SEN (95% CI)	SPE (95% CI)	AUC (95% CI)	SEN (95% CI)	SPE (95% CI)
**ADC**	0.711 (0.629–0.784)	0.680 (0.564–0.781)	0.723 (0.598–0.827)	0.676 (0.545–0.789)	0.677 (0.495–0.826)	0.643 (0.441–0.814)
**SER**	0.766 (0.688–0.833)	0.859 (0.762–0.927)	0.615 (0.486–0.733)	0.731 (0.603–0.836)	0.706 (0.525–0.849)	0.643 (0.441–0.814)
**Wash-in**	0.704 (0.622–0.777)	0.756 (0.646–0.847)	0.631 (0.502–0.747)	0.711 (0.629–0.819)	0.529 (0.351–0.702)	0.821 (0.631–0.939)
**Wash-out**	0.673 (0.590–0.749)	0.769 (0.660–0.857)	0.554 (0.425–0.677)	0.666 (0.535–0.781)	0.618 (0.436–0.778)	0.643 (0.441–0.814)
**Fusion 1**	0.804 (0.730–0.866)	0.769 (0.660–0.857)	0.739 (0.615–0.840)	0.770 (0.646–0.867)	0.941 (0.803–0.993)	0.536 (0.339–0.725)
**Fusion 2**	0.839 (0.768–0.895)	0.846 (0.747–0.918)	0.739 (0.615–0.840)	0.795 (0.674–0.887)	0.941 (0.803–0.993)	0.571 (0.372–0.755)

Fusion 1 represents the SVM model established by combining radiomics features from three DCE-MRI parameter maps (SER, wash-in, and wash-out). Fusion 2 represents the SVM model established by combining radiomics features from three DCE-MRI parameter maps and ADC maps.

AUC, area under the receiver operating characteristic curve; SEN, sensitivity; SPE, specificity; CI, confidential interval; SER, signal enhancement ratio; ADC, apparent diffusion coefficient.

**Table 4:** *p*-Values of DeLong’s test between SVM models.

Model	Cohort	ADC	SER	Wash-in	Wash-out	Fusion 1	Fusion 2
**ADC**	Training	/	0.204	0.892	0.507	0.027	0.002
	Validation	/	0.459	0.644	0.897	0.207	0.095
**SER**	Training	0.204	/	0.167	0.035	0.099	0.001
	Validation	0.459	/	0.784	0.399	0.242	0.114
**Wash-in**	Training	0.892	0.167	/	0.581	0.006	0.001
	Validation	0.644	0.784	/	0.615	0.377	0.219
**Wash-out**	Training	0.507	0.035	0.581	/	0.001	<0.001
	Validation	0.897	0.399	0.615	/	0.082	0.040
**Fusion 1**	Training	0.027	0.099	0.006	0.001	/	0.004
	Validation	0.207	0.242	0.377	0.082	/	0.340
**Fusion 2**	Training	0.002	0.001	0.001	<0.001	0.004	/
	Validation	0.095	0.114	0.219	0.040	0.340	/

Fusion 1 represents the SVM model established by combining radiomics features from three DCE-MRI parameter maps (SER, wash-in, and wash-out). Fusion 2 represents the SVM model established by combining radiomics features from three DCE-MRI parameter maps and ADC maps.

ADC, apparent diffusion coefficient; SER, signal enhancement ratio.

## Discussion

In this study, we explored whether the radiomics features of DCE-MRI parameter maps and ADC maps in patients with BC could be used to predict the preoperative Ki-67 proliferation index. The radiomics model constructed in this study performed well in identifying the low and high expression status of Ki-67.

Many previous studies have described Ki-67 expression, on the basis of IHC, as a prognostic and predictive indicator of BC. Higher Ki-67 expression status is associated with poorer response to treatment and poorer prognosis (33). The Ki-67 index can also play a role in distinguishing molecular subtypes of BC together with HER2 expression status (2). However, pathological biopsy only requires sampling in a part of the tumor tissue, so the Ki-67 proliferation index in the test results may not reflect tumor heterogeneity. With the development of imaging technology in recent years, imaging can provide a holistic picture of the anatomy and function of the tumor tissue. Therefore, imaging methods may be more convenient and may even provide more biological information for determining the expression status of Ki-67.

DCE-MRI is a highly sensitive method but has only moderate specificity for the diagnosis of invasive BC (34). In recent years, DWI with ADC maps has been increasingly used in multiparameter imaging environments for BC examination because it can be performed without a contrast agent (35–37). DWI can quantitatively measure the Brownian motion of free water in the tissue to provide functional information about the tissue structure and does not require intravenous injection (38). In addition, ADC has been found to increase the specificity of breast tumor diagnosis and complement DCE-MRI in tumor qualitative aspects (39, 40). In this study, we selected radiomics features extracted from DCE-MRI parameter maps and ADC maps and finally established six SVM classifier models. The ROC curve showed that the AUC score of the model combined with the parameter maps and ADC maps was higher than that of other single-parameter models in the training set and validation set. Therefore, the radiomics model including features of DCE-MRI parameter maps and ADC maps could improve the performance of Ki-67 expression status discrimination.

Radiomics, which provides potential biomarkers for clinical results through extracting and analyzing image features, is a relatively new technology (11). BC is highly heterogeneous. Compared with traditional genomics and proteomics, radiomics not only can non-invasively assess the tumor and its microenvironment but also can predict the genetic heterogeneity of the tumor (41). Herein, we used radiomics to quantitatively extract features within tumors on the basis of DCE-MRI parameters and ADC maps to reflect the heterogeneity of the internal tumor structure. A previous study has modeled radiomics features extracted from T2-weighted and contrast-enhanced T1-weighted images of BC and indicated that T2-weighted classifiers were important predictors of Ki-67 status (42). Li et al. (14) have combined peritumoral and intratumoral features from DCE-MRI functional parameter maps to determine Ki-67 status. Therefore, in this study, we established a multiparameter model based on the radiomics features of functional parametric maps and ADC maps. Our study achieved better predictive performance than the above multiparameter studies. Another multiparametric MRI study also using DWI sequences has achieved good performance in predicting Ki-67 status (24). However, these prior studies have analyzed only the largest tumor slices in two dimensions and therefore were unlikely to fully assess the heterogeneity of BC. The radiomics features that we extracted in this study were obtained from the three-dimensional volume model of the tumor, taking into account the interlayer correlation, and thus fully revealed the BC heterogeneity.

The current study had several limitations. First, the retrospective design of this study might have introduced inherent variations and biases. One source of variation was the changes in repetition time during DCE-MRI acquisition; however, the difference in MR signal intensity was not the principal factor in our study, because the radiomics features were derived from three parametric maps reflecting the changes in contrast medium concentration. Although the enhancement ratio is a function of repetition time, these functions change relatively slowly (43). Therefore, a well-designed prospective study supporting the results of this study is necessary. Second, this was an independent single-center study, and the number of patients included was limited. Therefore, the model established herein had several limitations, and datasets from other imaging units might have different spatial resolutions. Consequently, further research is required to verify the diagnostic performance of our model in a large multi-center patient sample. Finally, only semiquantitative parametric images were analyzed in this study. The application value of quantitative parametric images in radiomics will be further explored in future studies.

## Conclusion

In this study, our experimental results demonstrated that radiomics analysis based on DCE-MRI parameter maps and ADC maps can feasibly be used to predict the Ki-67 status in BC. Given that several different biomarkers must be integrated to make clinical management decisions for patients, our proposed model can be further extended in the future, such as by including more scanning sequences and predicting more molecular subtypes to support clinical decisions.

## Data availability statement

The raw data supporting the conclusions of this article will be made available by the authors, without undue reservation.

## Ethics statement

The studies involving human participants were reviewed and approved by Shengjing Hospital of China Medical University. The ethics committee waived the requirement of written informed consent for participation. Written informed consent was not obtained from the individual(s) for the publication of any potentially identifiable images or data included in this article.

## Author contributions

SF conducted data analysis and manuscript writing. JY was responsible for the manuscript revision. All authors critically reviewed and revised the manuscript. All authors contributed to the article and approved the submitted version.

## Funding

This study is supported by Research and development (R&D) foundation for major Science and Technology from Shenyang (No.19-112-4-105), Big data foundation for health care from China Medical University (No. HMB201902105) and Natural Fund Guidance Plan (No. 2019-ZD-0743).

## Conflict of interest

The authors declare that the research was conducted in the absence of any commercial or financial relationships that could be construed as a potential conflict of interest.

## Publisher’s note

All claims expressed in this article are solely those of the authors and do not necessarily represent those of their affiliated organizations, or those of the publisher, the editors and the reviewers. Any product that may be evaluated in this article, or claim that may be made by its manufacturer, is not guaranteed or endorsed by the publisher.

## References

[1] Bray F Ferlay J Soerjomataram I Siegel RL Torre LA Jemal A. Global cancer statistics 2018: GLOBOCAN estimates of incidence and mortality worldwide for 36 cancers in 185 countries. CA Cancer J Clin (2018) 68:394–424. doi: 10.3322/caac.21492 30207593

[2] Cheang MC Chia SK Voduc D Gao D Leung S Snider J. Ki67 index, HER2 status, and prognosis of patients with luminal b breast cancer. J Natl Cancer Inst (2009) 101:736–50. doi: 10.1093/jnci/djp082 PMC2684553 19436038

[3] Yerushalmi R Woods R Ravdin PM Hayes MM Gelmon KA. Ki67 in breast cancer: prognostic and predictive potential. Lancet Oncol (2010) 11:174–83. doi: 10.1016/S1470-2045(09)70262-1 20152769

[4] de Azambuja E Cardoso F de Castro G Jr. Colozza M Mano MS Durbecq V. Ki-67 as prognostic marker in early breast cancer: a meta-analysis of published studies involving 12,155 patients. Br J Cancer (2007) 96:1504–13. doi: 10.1038/sj.bjc.6603756 PMC2359936 17453008

[5] Healey MA Hirko KA Beck AH Collins LC Schnitt SJ Eliassen AH. Assessment of Ki67 expression for breast cancer subtype classification and prognosis in the nurses' health study. Breast Cancer Res Treat (2017) 166:613–22. doi: 10.1007/s10549-017-4421-3 PMC6995281 28791482

[6] Urruticoechea A Smith IE Dowsett M. Proliferation marker ki-67 in early breast cancer. J Clin Oncol (2005) 23:7212–20. doi: 10.1200/JCO.2005.07.501 16192605

[7] Inwald EC Klinkhammer-Schalke M Hofstadter F Zeman F Koller M Gerstenhauer M. Ki-67 is a prognostic parameter in breast cancer patients: results of a large population-based cohort of a cancer registry. Breast Cancer Res Treat (2013) 139:539–52. doi: 10.1007/s10549-013-2560-8 PMC3669503 23674192

[8] Stuart-Harris R Caldas C Pinder SE Pharoah P. Proliferation markers and survival in early breast cancer: A systematic review and meta-analysis of 85 studies in 32,825 patients. Breast (2008) 17:323–34. doi: 10.1016/j.breast.2008.02.002 18455396

[9] Carriero A Di Credico A Mansour M Bonomo L. Maximum intensity projection analysis in magnetic resonance of the breast. J Exp Clin Cancer Res (2002) 21:77–81. doi: 10.1200/JCO.2002.99.145 12585659

[10] Gillies RJ KP Hricak H. Radiomics: Images are more than pictures, they are data. Radiology (2016) 278:563–77. doi: 10.1148/radiol.2015151169 PMC4734157 26579733

[11] Lambin P Leijenaar RTH Deist TM Peerlings J de Jong EEC van Timmeren J. Radiomics: The bridge between medical imaging and personalized medicine. Nat Rev Clin Oncol (2017) 14:749–62. doi: 10.1038/nrclinonc.2017.141 28975929

[12] Choi BB. Dynamic contrast enhanced-MRI and diffusion-weighted image as predictors of lymphovascular invasion in node-negative invasive breast cancer. World J Surg Oncol (2021) 19:76. doi: 10.1186/s12957-021-02189-3 33722246 PMC7962354

[13] Bickelhaupt S Paech D Kickingereder P Steudle F Lederer W Daniel H. Prediction of malignancy by a radiomic signature from contrast agent-free diffusion MRI in suspicious breast lesions found on screening mammography. J Magn Reson Imaging (2017) 46:604–16. doi: 10.1002/jmri.25606 28152264

[14] Li C Song L Yin J. Intratumoral and peritumoral radiomics based on functional parametric maps from breast DCE-MRI for prediction of HER-2 and ki-67 status. J Magn Reson Imaging (2021) 54:703–14. doi: 10.1002/jmri.27651 33955619

[15] Dong Y Feng Q Yang W Lu Z Deng C Zhang L. Preoperative prediction of sentinel lymph node metastasis in breast cancer based on radiomics of T2-weighted fat-suppression and diffusion-weighted MRI. Eur Radiol (2018) 28:582–91. doi: 10.1007/s00330-017-5005-7 28828635

[16] Bickelhaupt S Jaeger PF Laun FB Lederer W Daniel H Kuder TA. Radiomics based on adapted diffusion kurtosis imaging helps to clarify most mammographic findings suspicious for cancer. Radiology (2018) 287:761–70. doi: 10.1148/radiol.2017170273 29461172

[17] Zhang Q Peng Y Liu W Bai J Zheng J Yang X. Radiomics based on multimodal MRI for the differential diagnosis of benign and malignant breast lesions. J Magn Reson Imaging (2020) 52:596–607. doi: 10.1002/jmri.27098 32061014

[18] Jiang Z Song L Lu H Yin J. The potential use of DCE-MRI texture analysis to predict HER2 2+ status. Front Oncol (2019) 9:242. doi: 10.3389/fonc.2019.00242 31032222 PMC6473324

[19] Song L Lu H Yin J. Preliminary study on discriminating HER2 2+ amplification status of breast cancers based on texture features semi-automatically derived from pre-, post-contrast, and subtraction images of DCE-MRI. PloS One (2020) 15:e0234800. doi: 10.1371/journal.pone.0234800 32555662 PMC7299320

[20] Molinari C Clauser P Girometti R Linda A Cimino E Puglisi F. MR mammography using diffusion-weighted imaging in evaluating breast cancer: A correlation with proliferation index. Radiol Med (2015) 120:911–8. doi: 10.1007/s11547-015-0527-z 25776017

[21] Zhang Y Zhu Y Zhang K Liu Y Cui J Tao J. Invasive ductal breast cancer: preoperative predict ki-67 index based on radiomics of ADC maps. Radiol Med (2020) 125:109–16. doi: 10.1007/s11547-019-01100-1 31696388

[22] Liu C Ding J Spuhler K Gao Y Serrano Sosa M Moriarty M. Preoperative prediction of sentinel lymph node metastasis in breast cancer by radiomic signatures from dynamic contrast-enhanced MRI. J Magn Reson Imaging (2019) 49:131–40. doi: 10.1002/jmri.26224 PMC6298835 30171822

[23] Shin JK Kim JY. Dynamic contrast-enhanced and diffusion-weighted MRI of estrogen receptor-positive invasive breast cancers: Associations between quantitative MR parameters and ki-67 proliferation status. J Magn Reson Imaging (2017) 45:94–102. doi: 10.1002/jmri.25348 27313102

[24] Jiang T Song J Wang X Niu S Zhao N Dong Y. Intratumoral and peritumoral analysis of mammography, tomosynthesis, and multiparametric MRI for predicting ki-67 level in breast cancer: a radiomics-based study. Mol Imaging Biol (2022) 24:550–9. doi: 10.1007/s11307-021-01695-w 34904187

[25] Eun NL Kang D Son EJ Park JS Youk JH Kim JA. Texture analysis with 3.0-T MRI for association of response to neoadjuvant chemotherapy in breast cancer. Radiology (2020) 294:31–41. doi: 10.1148/radiol.2019182718 31769740

[26] Lu H Yin J. Texture analysis of breast DCE-MRI based on intratumoral subregions for predicting HER2 2+ status. Front Oncol (2020) 10:543. doi: 10.3389/fonc.2020.00543 32373531 PMC7186477

[27] Hammond ME Hayes DF Dowsett M Allred DC Hagerty KL Badve S. American Society of clinical Oncology/College of American pathologists guideline recommendations for immunohistochemical testing of estrogen and progesterone receptors in breast cancer. J Clin Oncol (2010) 28:2784–95. doi: 10.1200/JCO.2009.25.6529 PMC2881855 20404251

[28] Goldhirsch A Wood WC Coates AS Gelber RD Thurlimann B Senn HJ. Strategies for subtypes–dealing with the diversity of breast cancer: Highlights of the st. gallen international expert consensus on the primary therapy of early breast cancer 2011. Ann Oncol (2011) 22:1736–47. doi: 10.1093/annonc/mdr304 PMC3144634 21709140

[29] Wolff AC Hammond ME Hicks DG Dowsett M McShane LM Allison KH. Recommendations for human epidermal growth factor receptor 2 testing in breast cancer: American society of clinical Oncology/College of American pathologists clinical practice guideline update. J Clin Oncol (2013) 31:3997–4013. doi: 10.1200/JCO.2013.50.9984 24101045

[30] Fan M Yuan W Zhao W Xu M Wang S Gao X. Joint prediction of breast cancer histological grade and ki-67 expression level based on DCE-MRI and DWI radiomics. IEEE J BioMed Health Inform (2020) 24:1632–42. doi: 10.1109/JBHI.2019.2956351 31794406

[31] van Griethuysen JJM Fedorov A Parmar C Hosny A Aucoin N Narayan V. Computational radiomics system to decode the radiographic phenotype. Cancer Res (2017) 77:e104–7. doi: 10.1158/0008-5472.CAN-17-0339 PMC5672828 29092951

[32] Liu Z Zhang XY Shi YJ Wang L Zhu HT Tang Z. Radiomics analysis for evaluation of pathological complete response to neoadjuvant chemoradiotherapy in locally advanced rectal cancer. Clin Cancer Res (2017) 23:7253–62. doi: 10.1158/1078-0432.CCR-17-1038 28939744

[33] Jones RL Salter J A'Hern R Nerurkar A Parton M Reis-Filho JS. The prognostic significance of Ki67 before and after neoadjuvant chemotherapy in breast cancer. Breast Cancer Res Treat (2009) 116:53–68. doi: 10.1007/s10549-008-0081-7 18592370

[34] Warren RM Pointon L Thompson D Hoff R Gilbert FJ Padhani A. Reading protocol for dynamic contrast-enhanced MR images of the breast: Sensitivity and specificity analysis. Radiology (2005) 236:779–88. doi: 10.1148/radiol.2363040735 16118160

[35] Ramalho J Semelka RC Ramalho M Nunes RH AlObaidy M Castillo M. Gadolinium-based contrast agent accumulation and toxicity: An update. AJNR Am J Neuroradiol (2016) 37:1192–8. doi: 10.3174/ajnr.A4615 PMC7960350 26659341

[36] Rotili A Trimboli RM Penco S Pesapane F Tantrige P Cassano E. Double reading of diffusion-weighted magnetic resonance imaging for breast cancer detection. Breast Cancer Res Treat (2020) 180:111–20. doi: 10.1007/s10549-019-05519-y 31938940

[37] Spick C Bickel H Pinker K Bernathova M Kapetas P Woitek R. Diffusion-weighted MRI of breast lesions: A prospective clinical investigation of the quantitative imaging biomarker characteristics of reproducibility, repeatability, and diagnostic accuracy. NMR BioMed (2016) 29:1445–53. doi: 10.1002/nbm.3596 27553252

[38] Partridge SC Nissan N Rahbar H Kitsch AE Sigmund EE. Diffusion-weighted breast MRI: Clinical applications and emerging techniques. J Magn Reson Imaging (2017) 45:337–55. doi: 10.1002/jmri.25479 PMC5222835 27690173

[39] Zhang L Tang M Min Z Lu J Lei X Zhang X. Accuracy of combined dynamic contrast-enhanced magnetic resonance imaging and diffusion-weighted imaging for breast cancer detection: a meta-analysis. Acta Radiol (2016) 57:651–60. doi: 10.1177/0284185115597265 26275624

[40] Mendez CA Pizzorni Ferrarese F Summers P Petralia G Menegaz G. DCE-MRI and DWI integration for breast lesions assessment and heterogeneity quantification. Int J BioMed Imaging (2012) 2012:676808. doi: 10.1155/2012/676808 23213317 PMC3507154

[41] Joseph C Papadaki A Althobiti M Alsaleem M Aleskandarany MA Rakha EA. Breast cancer intratumour heterogeneity: current status and clinical implications. Histopathology (2018) 73:717–31. doi: 10.1111/his.13642 29722058

[42] Liang C Cheng Z Huang Y He L Chen X Ma Z. An MRI-based radiomics classifier for preoperative prediction of ki-67 status in breast cancer. Acad Radiol (2018) 25:1111–7. doi: 10.1016/j.acra.2018.01.006 29428211

[43] Chen PC Lin DJ Jao JC Hsiao CC Lin LM Pan HB. The impact of flip angle and TR on the enhancement ratio of dynamic gadobutrol-enhanced MR imaging: *In vivo* VX2 tumor model and computer simulation. Magn Reson Med Sci (2015) 14:193–202. doi: 10.2463/mrms.2014-0048 25833269

