# Supplementary material for: Radiomics of dynamic contrast-enhanced magnetic resonance imaging parametric maps and apparent diffusion coefficient maps to predict Ki-67 status in breast cancer
Source: Front Oncol. 2022 Nov 25;12:847880. doi: 10.3389/fonc.2022.847880 (PMC9989944; doi:10.3389/fonc.2022.847880)
Supplement: Supplementary file 1 [file Table_1.docx]

**Supplementary Table S1.** Feature description

| Image types | Feature group | Quantity | Description |
| --- | --- | --- | --- |
| Original | Shape | 8 | 8 descriptors (Voxel Volume, Surface Area, Surface Volume Ratio, Sphericity, Maximum 3D Diameter, Spherical Disproportion, Compactness1, Compactness2) contain descriptors of the three-dimensional size and shape of the ROI. |
| Original  Wavelet  LoG (sigma [3.0, 5.0 mm]) | First order | 19 | 19 descriptors (Energy, Total Energy, Entropy, Minimum, 10th percentile, 90th percentile, Maximum, Mean, Median, Interquartile Range, Range, Mean Absolute Deviation, Robust Mean Absolute Deviation, Root Mean Squared, Standard Deviation, Kurtosis, Variance, Uniformity) describe the distribution of voxel intensities within the image region defined by the mask through commonly used and basic metrics. |
|  | Gray Level Co-occurrence Matrix (GLCM) | 22 | 22 descriptors (Autocorrelation, Joint Average, Cluster Prominence, Cluster Shade, Cluster Tendency, Contrast, Correlation, Difference Average, Difference Entropy, Difference Variance, Joint Energy, Joint Entropy, Imc1, Imc2, Idm, Idmn, Id, Idn, Inverse Variance, Maximum Probability, Sum Entropy, Sum Squares) describes the second-order joint probability function of an image region constrained by the mask. |
|  | Gray Level Size Zone Matrix (GLSZM) | 16 | 16 descriptors (Small Area Emphasis, Large Area Emphasis, Gray Level Non-Uniformity, Gray Level Non-Uniformity Normalized, Size-Zone Non-Uniformity, Size-Zone Non-Uniformity Normalized, Zone Percentage, Gray Level Variance, Zone Variance, Zone Entropy, Low Gray Level Zone Emphasis, High Gray Level Zone Emphasis, Small Area Low Gray Level Emphasis, Small Area High Gray Level Emphasis, Large Area Low Gray Level Emphasis, Large Area High Gray Level Emphasis)  quantify gray level zones in an image. A gray level zone is defined as the number of connected voxels that share the same gray level intensity |
|  | Gray Level Run Length Matrix (GLRLM) | 16 | 16 descriptors (Short Run Emphasis,  Long Run Emphasis, Gray Level Non-Uniformity, Gray Level Non-Uniformity Normalized, Run Length Non-Uniformity, Run Length Non-Uniformity Normalized,  Run Percentage, Gray Level Variance, Run Variance, Run Entropy, Low Gray Level Run Emphasis, High Gray Level Run Emphasis, Short Run Low Gray Level Emphasis, Short Run High Gray Level Emphasis, Long Run Low Gray Level Emphasis, Long Run High Gray Level Emphasis ) quantify gray level runs, which are defined as the length in number of pixels, of consecutive pixels that have the same gray level value. |
|  | Gray Level Dependence Matrix (GLDM) | 14 | 14 descriptors（Small Dependence Emphasis, Large Dependence Emphasis, Gray Level Non-Uniformity, Dependence Non-Uniformity, Dependence Non-Uniformity Normalized, Gray Level Variance, Dependence Variance, Dependence Entropy, Low Gray Level Emphasis, High Gray Level Emphasis, Small Dependence Low Gray Level Emphasis, Small Dependence High Gray Level Emphasis, Large Dependence Low Gray Level Emphasis, Large Dependence High Gray Level Emphasis）quantify gray level dependencies in an image. |

NOTE. LoG, Laplacian of Gaussian.

**Supplementary Table S2.** Description of the selected radiomics features from three DCE-MRI parameter maps

| Different map | Radiomic feature | Radiomic group | Feature class filter |
| --- | --- | --- | --- |
| SER | Dependence Non Uniformity Normalized | Gldm | Log-Sigma-3.0mm |
| SER | Dependence Variance | Gldm | Log-Sigma-3.0mm |
| SER | Minimum | Firstorder | Log-sigma-5.0mm |
| SER | 10 Percentile | Firstorder | Wavelet-LLH |
| SER | Run Variance | Glrlm | Wavelet-LHL |
| SER | Autocorrelation | Glcm | Wavelet-LHH |
| Wash in | Minimum | Firstorder | Log-sigma-3.0mm |
| Wash in | Large Area High Gray Level Emphasis | Glszm | Log-sigma-5.0mm |
| Wash in | Range | Firstorder | Wavelet-LHH |
| Wash in | Small Dependence High Gray Level Emphasis | Gldm | Wavelet-LHH |
| Wash in | Large Dependence Low Gray Level Emphasis | Gldm | Wavelet-HHL |
| Wash out | Size Zone Non Uniformity Normalized | Glszm | Log-sigma-3.0mm |
| Wash out | Correlation | Glcm | Log-sigma-3.0mm |
| Wash out | Joint Energy | Glcm | Wavelet-LLL |

NOTE. SER, signal enhancement ratio; Gldm, gray-level dependence matrix; Glrlm, gray-level run length matrix; Glcm, gray-level co-occurrence matrix; Glszm, gray-level size zone matrix; LoG, Laplacian of Gaussian.

**Supplementary Table S3.** Description of the selected radiomics features from SER maps

| Different map | Radiomic feature | Radiomic group | Feature class filter |
| --- | --- | --- | --- |
| SER | High Gray Level Run Emphasis | Glrlm | Original |
| SER | Dependence Non Uniformity Normalized | Gldm | Log-Sigma-3.0mm |
| SER | Range | Firstorder | Log-sigma-5.0mm |
| SER | Difference Entropy | Glcm | Log-sigma-5.0mm |
| SER | Small Area Low Gray Level Emphasis | Glszm | Log-sigma-5.0mm |
| SER | Kurtosis | Firstorder | Wavelet-HLL |

NOTE. SER, signal enhancement ratio; Gldm, gray-level dependence matrix; Glrlm, gray-level run length matrix; Glcm, gray-level co-occurrence matrix; Glszm, gray-level size zone matrix; LoG, Laplacian of Gaussian.

**Supplementary Table S4.** Description of the selected radiomics features from wash in maps

| Different map | Radiomic feature | Radiomic group | Feature class filter |
| --- | --- | --- | --- |
| Wash in | Imc1 | Glcm | Log-sigma-3.0mm |
| Wash in | Dependence Non Uniformity Normalized | Gldm | Log-sigma-3.0mm |
| Wash in | Small Area Low Gray Level Emphasis | Glszm | Log-sigma-5.0mm |
| Wash in | Range | Firstorder | Wavelet-LHH |

NOTE. Gldm, gray-level dependence matrix; Glcm, gray-level co-occurrence matrix; Glszm, gray-level size zone matrix; LoG, Laplacian of Gaussian.

**Supplementary Table S5.** Description of the selected radiomics features from wash out maps

| Different map | Radiomic feature | Radiomic group | Feature class filter |
| --- | --- | --- | --- |
| Wash out | Correlation | Glcm | Log-Sigma-3.0mm |
| Wash out | Size Zone Non Uniformity Normalized | Glszm | Log-sigma-3.0mm |
| Wash out | Denpendence Entropy | Gldm | Log-sigma-3.0mm |
| Wash out | Low Gray Level Zone Emphasis | Glszm | Wavelet-LLH |

NOTE. Gldm, gray-level dependence matrix; Glcm, gray-level co-occurrence matrix; Glszm, gray-level size zone matrix; LoG, Laplacian of Gaussian.
